# Supplementary material for: Trajectories of the Prevalence of Sarcopenia in the Pre- and Post-Stroke Periods: A Systematic Review
Source: Nutrients. 2022 Dec 26;15(1):113. doi: 10.3390/nu15010113 (PMC9824538; doi:10.3390/nu15010113)
Supplement: Supplementary file 1 [file nutrients-15-00113-s001.zip › nutrients-2102865-supplementary.pdf]

Table S1. Search strategy for a systematic review.

**MEDLINE**	
S1	(MH "Sarcopenia")
S2	(MH "Muscular Atrophy+")
S3	AB (sarcopeni* OR "muscul* atroph*" OR "muscul* wasting*" OR "muscul* weak* " OR "muscul* loss*") OR TI (sarcopeni* OR "muscul* atroph*" OR "muscul* wasting*" OR "muscul* weak* " OR "muscul* loss*")
S4	(MH "Stroke+")
S5	(MH "Hemorrhagic Stroke")
S6	(MH "Cerebrovascular Disorders+")
S7	(MH "Brain Infarction")
S8	(MH "Ischemic Stroke+")
S9	AB (hemorrhagic stroke* OR Cerebrovascular Disorder* OR Brain Infarction* OR Ischemic Stroke* OR Ischaemic Stroke*) OR TI (hemorrhagic stroke* OR Cerebrovascular Disorder* OR Brain Infarction* OR Ischemic Stroke* OR Ischaemic Stroke*)
S10	S1 OR S2 OR S3
S11	S4 OR S5 OR S6 OR S7 OR S8
S12	S9 OR S11
S13	S10 AND S12
S14	(MH "Animals+")
S15	(MH "Humans")
S16	S14 NOT S15
S17	S13 NOT S16
EMBASE	
S1	EMB.EXACT.EXPLODE("sarcopenia")
S2	(EMB.EXACT.EXPLODE("muscle atrophy"))
S3	ab(sarcopeni* OR "muscul* atroph*" OR "muscul* wasting*" OR "muscul* weak* " OR "muscul* loss*") OR ti(sarcopeni* OR "muscul* atroph*" OR "muscul* wasting*" OR "muscul* weak* " OR "muscul* loss*")
S4	(EMB.EXACT.EXPLODE("cerebrovascular accident")) OR (EMB.EXACT.EXPLODE("brain hemorrhage")) OR (EMB.EXACT.EXPLODE("cerebrovascular disease")) OR (EMB.EXACT.EXPLODE("brain infarction")) OR (EMB.EXACT.EXPLODE("ischemic stroke"))
S5	("hemorrhagic stroke*" OR "Cerebrovascular Disorder*" OR "Brain Infarction*" OR "Ischemic Stroke*" OR "Ischaemic Stroke*") OR ti("hemorrhagic stroke*" OR "Cerebrovascular Disorder*" OR "Brain Infarction*" OR "Ischemic Stroke*" OR "Ischaemic Stroke*")
S6	(EMB.EXACT.EXPLODE("animal") or EMB.EXACT.EXPLODE("animal experiment")) NOT (EMB.EXACT.EXPLODE("human") OR EMB.EXACT.EXPLODE("human experiment"))
S7	S3 OR S2 OR S1
S8	S5 OR S4
S9	S8 AND S7
S10	S9 NOT S6
S11	S10 AND PD(20100401-20220430)
S12	S11 NOT DTYPE(abstract report OR conference abstract)
Web of Science	

#1	TS=(sarcopeni* OR "muscul* atroph*" OR "muscul* wasting*" OR "muscul* weak* " OR "muscul* loss*")
#2	TS=(hemorrhagic stroke* OR Cerebrovascular Disorder* OR Brain Infarction* OR Ischemic Stroke* OR Ischaemic Stroke*)
#3	TS=(animal not human)
#4	#1 AND #2
#5	#4 NOT #3
#6	#5 and DOP=(2010-04-01 to 2022-04-30)
#7	#6 and Meeting Abstracts (Exclude – Document Types) and DOP=(2010-04-01 to 2022-04-30)
Central Register of Controlled Trials	
S1	SU Sarcopenia
S2	SU Muscular Atrophy
S3	TI (sarcopeni* OR muscul* atroph* OR muscul* wasting* OR muscul* weak* OR muscul* loss*) OR AB (sarcopeni* OR muscul* atroph* OR muscul* wasting* OR muscul* weak* OR muscul* loss*)
S4	SU Stroke
S5	SU Hemorrhagic Stroke
S6	SU Cerebrovascular Disorders
S7	SU Brain Infarction
S8	SU Ischemic Stroke
S9	TI (hemorrhagic stroke* OR Cerebrovascular Disorder* OR Brain Infarction* OR Ischemic Stroke* OR Ischaemic Stroke*)) OR AB (hemorrhagic stroke* OR Cerebrovascular Disorder* OR Brain Infarction* OR Ischemic Stroke* OR Ischaemic Stroke*）
S10	S1 OR S2 OR S3
S11	S4 OR S5 OR S6 OR S7 OR S8 OR S9
S12	S10 AND S11
CINAHL	
S1	(MH "Sarcopenia") OR (MH "Muscular Atrophy+") OR (TI (sarcopeni* OR "muscul* atroph*" OR "muscul* wasting*" OR "muscul* weak* " OR "muscul* loss*") OR AB (sarcopeni* OR "muscul* atroph*" OR "muscul* wasting*" OR "muscul* weak* " OR "muscul* loss*"))
S2	(MH "Stroke+") OR (MH "Hemorrhagic Stroke") OR (MH "Cerebrovascular Disorders+") OR (MH "Infarction+") OR (MH "Ischemic Stroke+") OR (TI ((hemorrhagic stroke* OR Cerebrovascular Disorder* OR Brain Infarction* OR Ischemic Stroke* OR Ischaemic Stroke*)) OR AB ((hemorrhagic stroke* OR Cerebrovascular Disorder* OR Brain Infarction* OR Ischemic Stroke* OR Ischaemic Stroke*)))
S3	(MH "Animals+") NOT (MH "Human")
S4	S1 AND S2
S5	S5 NOT S4
医中誌Web	
#1	筋肉減少症/TH
#2	筋萎縮症/TH
#3	筋力低下/TA or 筋消耗/TA or 筋委縮/TA or 筋力減退/TA or 筋量減少/TA or 筋肉減弱/TA or サルコペニア/TA
#4	脳卒中/TH

#5	出血性卒中/TA
#6	脳血管発作/TA
#7	脳血管障害/TH
#8	脳梗塞/TH
#9	脳血管障害/TA
#10	脳梗塞/TA
#11	虚血性脳梗塞/TA
#12	脳卒中/TA
#13	#1 or #2 or #3
#14	#4 or #5 or #6 or #7 or #8 or #9 or #10 or #11 or #12
#15	#13 and #14
#16	(#15) and (PT=会議録除く CK=ヒト PDAT=2010/04/01:2022/04/30)

Table S2. Summary of the included studies.

Author Year Country	Study design	Time from onset to stroke	Setting	Age (years)	Sample size Male/Female	Diagnostic criteria	Measurement of muscle mass/muscle strength/ physical performance	Sarcopenia prevalence, %
Yao et al. 2022 [53] China	Cross-sectional study	Mean 67 days	Not stated	Mean 57	364<br>270/94	AWGS2019<br>Muscle mass<br>Muscle strength<br>Physical performance	BIA/<br>Grip strength/<br>Gait speed<br>(Cutoff: ≤ 0.8 m/s)	49.5%
Kanai et al. 2022 [17] Japan	Cross-sectional study	Length of acute hospital stay:<br>mean 25.5 (IQR: 20.0–31.8) days	Convalescent rehabilitation hospital	Median 72.0<br>(IQR: 61.3–82.8)	80<br>38/42	AWGS2019<br>Muscle mass<br>Muscle strength<br>Physical performance	BIA/<br>Grip strength/<br>SPPB	57.5%
Shimizu et al. 2022 [18] Japan	Cross-sectional study	IDDSI framework levels<br>Level 7: 21 (IQR: 14–30) days<br>Level 6: 25 (19–36) days<br>Level 5: 29 (19–37) days<br>Level 4: 37 (24–50) days<br>Level 3: 44 (39–54) days	Convalescent rehabilitation hospital	Mean<br>77.5 ± 7.7	443<br>244/199	EWGSOP2<br>Muscle strength<br>Muscle mass<br>(Cutoff: AWGS2019)	BIA/<br>Grip strength	62.1%
Sato et al. 2022 [12] Japan	Retrospective observational study	Within 5 days after admission	Acute care hospital	Median 74<br>(IQR: 65–83)	211<br>74/147	AWGS2019<br>Muscle mass<br>Muscle strength	BIA/<br>Grip strength	30.8%
Shimizu et al. 2021 [19] Japan	Prospective observational cohort study (All patients are patients with dysphagia)	Delayed Dysphagia group:<br>median 22 (IQR: 12–33) days<br>Early Dysphagia group:<br>27 (19–39) days	Convalescent rehabilitation hospital	Mean<br>79.1 ± 8.0	165<br>77/88	AWGS2019<br>Muscle mass<br>Muscle strength	BIA/<br>Grip strength	73.9%

Shiraishi et al. 2021 [20] Japan	Retrospective cohort study	Median 13 (IQR: 10–22) days	Convalescent rehabilitation hospital	Mean 72.0 ± 13.5	300 155/145	AWGS2019 Muscle mass Muscle strength	BIA/ Grip strength	49.7%
Choi et al. 2021 [54] Korea	Cross-sectional study	Sarcopenia group: mean 19.77 ± 7.07 days Non-sarcopenia group: 26.85 ± 13.35 days	Subacute phase	Sarcopenia group: 69.79 ± 9.82 non-sarcopenia group: 65.38 ± 12.29	30 20/10	Foundation for the National Institutes of Health Sarcopenia Project	DEXA	46.7%
Nozoe et al. 2021 [13] Japan	Cross-sectional and longitudinal study	Within 5 days of admission	Acute care hospital	Mean 76 ± 7	289 163/126	SARC-F		19%
Yoshimura et al. 2021 [21] Japan	Retrospective cohort study	eGFR (mL/min/1.73 m^2) >90: median 12 (IQR: 10–23) days 60–89: 12 (9–20) days 30–59: 13 (11–21) days 15–29: 15 (11–28) days <15: 15 (12–15) days	Convalescent rehabilitation wards	Mean 73.5 ± 11.8	813 422/391	AWGS2019 Muscle mass Muscle strength	BIA/ Grip strength	47.4%
Nishioka et al. 2021 [22] Japan	Retrospective cohort study	Median 25 (IQR: 18–34) days	Convalescent rehabilitation wards	Mean 73.1 ± 12.5	408 238/170	AWGS2019 Muscle mass Muscle strength	BIA/ Grip strength	69%
Yoshimura et al. 2020 [23] Japan	Retrospective cohort study	Median 14 (IQR: 10–21) days	Convalescent rehabilitation wards	Mean 72.4 ± 13.6	598 315/283	AWGS2019 Muscle mass Muscle strength	BIA/ Grip strength	46.2%
Nagano et al. 2020 [24] Japan	Retrospective cohort study	Mean 17.5 ± 12.8 days	Convalescent rehabilitation wards	Mean 79.3 ± 9.9	272 70/50	AWGS2019 Muscle mass Muscle strength	BIA/ Grip strength	44%
Chang et al. 2020 [55] Taiwan	Cross-sectional design	Stroke onset from at least 6 months ago	Outpatient department	Mean 64.6 ± 15.1	37 23/14	EWGSOP Muscle mass	DEXA	48.6%
Aydin et al. 2021 [56] Turkey	Cross-sectional, prospective and	Median 14 (Range: 5–135) months	Chronic stroke patients	Mean 64.6 ± 9.1	81 41/40	EWGSOP2 Muscle mass Muscle strength Physical performance	BIA/ Grip strength/ Gait speed and SPPB	Low muscle mass /BMI: 16% SMI: 1.2%

	observational clinical trial		who underwent inpatient rehabilitation					
Nozoe et al. 2021 [33] Japan	Cross-sectional study	Pre-stroke	Acute care hospital	Mean 76 ± 7	223 127/96	SARC-F		15%
Abe et al. 2022 [14] Japan	Retrospective cohort study	Within 5 days of admission	Acute care hospital	Mean 73.9 ± 12.6	244 161/83	Muscle mass and phase angle	BIA	Low PhA + SMI: 8.6% Pre-sarcopenia: 22.5% Dynapenia: 4.1%
Nozoe et al. 2019 [34] Japan	Prospective cohort study	Pre-stroke	Acute care hospital	Mean 76 ± 11	152 81/71	SARC-F		18%
Yoshimura et al. 2019 [25] Japan	Retrospective cohort study	Mean 15 ± 5 days	Convalescent rehabilitation wards	Mean 74.9 ± 13.2	795 324/471	EWGSOP Muscle strength Muscle mass (Cutoff: AWGS)	BIA/ Grip strength	52.1%
Jang et al. 2020 [57] Korea	Retrospective cohort analysis	2 weeks	University-affiliated hospital	Mean 64.3 ± 13.0	194 115/79	EWGSOP2 Muscle strength (Cutoff: AWGS2019)	Grip strength	41.8%
Yoshimura et al. 2018 [26] Japan	Cross-sectional study	Mean 15 ± 5 days	Convalescent rehabilitation wards	Mean 74 ± 13	637 271/366	EWGSOP Muscle strength Muscle mass (Cutoff: AWGS)	BIA/ Grip strength	53.6%
Shiraishi et al. 2018 [27] Japan	Cross-sectional study	Median 12 (10–19) days	Convalescent rehabilitation wards	Mean 72 ± 12	202 107/95	EWGSOP Muscle strength Muscle mass (Cutoff: AWGS)	BIA/ Grip strength	53.5%
Ryan et al. 2017 [43] USA	Cohort study	>6 months after onset of stroke	A veterans affairs medical center and a university hospital	Mean 63 ± 1	190 61%/57%	ALM/BMI criteria (muscle mass) EWGSOP criteria (muscle mass and gait speed) IWG (muscle mass and gait speed)	DEXA/ Gait speed	ALM/BMI criteria: 17.9% EWGSOP criteria: 14.I% IWGS: 16.7%

						ALM/height^2 (muscle mass)		ALM/ht2: 16.8%
Park et al. 2014 [58] Korea	Cross-sectional study	Not stated	Non-institutionalized population	Male<br>50–54: 34.1%<br>55–59: 21.1%<br>60–64: 16.9%<br>65–69: 12.6%<br>70–: 15.1%<br>Female<br>50–54: 33.1%<br>55–59: 17.9%<br>60–64: 13.5%<br>65–69: 12.4%<br>70–: 22.8%	4611<br>2029/2582	Janssen et al. criteria	DEXA	Class I: 11.8%<br>Class II: 4.92%
Nozoe et al. 2019 [35] Japan	Cross-sectional study	Pre-stroke	Acute care hospital	Mean<br>75 ± 11	183<br>103/80	SARC-F		15%
Maeda et al. 2017 [59] Japan	Cross-sectional study	Within 5 days of admission	Geriatric hospital,<br>acute care hospital	Mean<br>83.0 ± 8.2	619<br>252/367	AWGS<br>Muscle mass<br>Muscle strength	BIA/<br>Grip strength	63.6%
Lee et al. 2022 [15] Korea	Retrospective observational study	From symptom onset to diagnosis:<br>4.2 ± 2.6 days	Acute care hospital	Mean<br>65.5 ± 12.6	568<br>367/201	AWGS2019<br>Muscle mass<br>Muscle strength	BIA/<br>Medical Research Council (score)	8.5%
Inoue et al. 2021 [28] Japan	Cross-sectional study	Median 29<br>(IQR: 22–39) days	Convalescent rehabilitation wards	Mean<br>76.6 ± 7.5	256<br>146/110	AWGS2019<br>Muscle mass<br>Muscle strength	BIA/<br>Grip strength	63.7 %
Matsushita et al. 2019 [29] Japan	Retrospective study	Median 24<br>(IQR: 18–33) days	Convalescent rehabilitation wards	Mean<br>72.5 ± 13.2	267<br>150/117	EWGSOP2<br>Muscle mass<br>Muscle strength	BIA/<br>Grip strength	48.3%
Bellelli et al. 2018 [16] Italy	Cross-sectional study	First 24 h following admission	Acute geriatric units	Mean<br>80.9 ± 6.8	588<br>275/313	EWGSOP2<br>Muscle mass<br>Muscle strength<br>Physical performance	BIA/<br>Grip strength/<br>Gait speed	33.8%
Su et al. 2021 [36] Japan	Prospective observational study	Pre-stroke	Acute hospital	Mean<br>68.5 ±10.0	95<br>59/36	SARC-F		8%

Tanaka et al. (Japanese) 2021 [30] Japan	Retrospective cohort study	Not stated	Convalescent rehabilitation wards	Mean 72. 8 ± 11.9	69 34/35	AWGS2019 Muscle mass Muscle strength	BIA/ Grip strength	35%
Shibasaki et al. (Japanese) 2021 [60] Japan	Retrospective cohort study	Mean 0.6 ± 0.7 days	Acute hospital	Mean 77.0 ± 11.7	101 64/37	AWGS2019 Muscle mas Muscle strength	BIA/ Grip strength	57%
Ito et al. (Japanese) 2021 [31] Japan	Retrospective cohort study	A study that included patients who were not originally sarcopenic and calculated the incidence of sarcopenia at discharge from the hospital	Convalescent rehabilitation wards	Mean 63.7 ± 12.5	147 99/48	AWGS2019 Muscle mass Muscle strength Physical performance	BIA/ Grip strength/ Gait speed	New onset of sarcopenia: 9.5%
Tanaka et al. (Japanese) 2021 [61] Japan	Not stated	At admission and 2 weeks after admission	Acute hospital	72 (66-84)	22 13/9	AWGS2019 Muscle mass Muscle strength	CC/ Grip strength	At admission: 50.0% After 2 weeks: 45.5%
Yoshimura et al. 2018 [32] Japan	Retrospective cohort study	Median 14 (IQR: 9–19) days	Convalescent rehabilitation wards	Mean 74.1 ± 8.2	204 109/95	AWGS2019 Muscle mass Muscle strength	BIA/ Grip strength	39.7%

Abbreviations: AWGS, Asian Working Group for Sarcopenia; BIA, Bioimpedance Analysis; SPPB, Short Physical Performance Battery; IDDSI, International Dysphagia Diet Standardization Initiative; EWGSOP, European Working Group on Sarcopenia in Older People; DEXA, Dual-Energy X-ray Absorptiometry; eGFR, estimated Glomerular Filtration Rate; BMI, Body Mass Index; SMI, Skeletal Muscle Index; PhA, Phase Angle; ALM, Appendicular Lean Mass; IWG, International Working Group on Sarcopenia; CC, Calf circumference.
